# Long intergenic non-coding RNA 00511 (LINC00511) genetic variations and haplotype implication for colorectal cancer susceptibility and prognosis

**DOI:** 10.1038/s41598-025-10938-7

**Published:** 2025-08-11

**Authors:** Eman F. Sanad, Ahmad A. Hady, Mohamed Ali, Shorouk Eldash, Nermeen H. Elmorshedy, Farah Ayman, Hams M. Khattab, Amr Maher, Hadeel Ashree, Mahitab Abdelhady, Mazen Mohamed, Ahmed Adel, Sajed Khalil, Omar Alyan, Ahmed Samir, Alhassan A. Bakr, Nadia M. Hamdy

**Affiliations:** 1 Biochemistry Department, Faculty of Pharmacy, Ain Shams University, Abassia, Cairo, 11566 Egypt; 2 Department of Clinical Oncology and Nuclear Medicine, Faculty of Medicine, Mansoura University, Mansoura, 35516 Egypt; 3 Clinical Pharmacy Department, Faculty of Pharmacy, Ain Shams University, Abassia, Cairo, 11566 Egypt; 4 Department of Medicine, University of Chicago, Chicago, IL 60637 USA; 5 Pharmacology and Biochemistry Department, Faculty of Pharmacy, The British University in Egypt (BUE), ElSherouk, Cairo, 11837 Egypt; 6 Drug Design Program Graduation Project Students, Faculty of Pharmacy, Ain Shams University, Abassia, Cairo, 11566 Egypt

**Keywords:** Long intergenic non-coding RNA 005 **(**LINC00511), Single nucleotide polymorphism (SNPs), Colorectal cancer (CRC), Biochemistry, Cancer, Molecular biology

## Abstract

Long intergenic non-coding RNA 00,511 (LINC00511) is considered an oncogene for various cancers. However, the association between LINC00511 single nucleotide polymorphisms (SNPs) and colorectal cancer (CRC) remains unclear. Our study aims to study whether LINC00511 SNPs could predict CRC susceptibility or prognosis, an important step-toward precision-health, based on an Egyptian CRC patient cohort. A total of 200 CRC patients and 200 cancer-free controls were genotyped for three LINC00511 SNPs − rs9906859, rs1558535, and rs17780195 using qRT-PCR. Studied SNPs were in strong linkage disequilibrium and moderately correlated in all groups. Genotype association concerning tumor stage, revealed rs1558535 AT and rs17780195 AG variants correlated significantly with CRC advanced stages (adjusted OR: 3.99 and 2.72), respectively. Logistic regression showed that rs1558535 and rs9906859 genotypes were associated with CRC. Haplotype analysis disclosed that ‘T_rs155535_A_rs17780195_C_rs9906859_’ mutant-wild-mutant haplotype has 1.5-fold increased CRC risk (OR: 1.46, 95% CI: 1.07–1.99). ‘T_rs155535_A_rs17780195_T_rs9906859_’ haplotype conferred fivefold lower CRC risk (OR: 0.20, 95% CI: 0.09–0.47). Epistasis analysis showed individuals heterozygote and homozygote or homozygote and heterozygote for rs1558535 and rs9906859 are at high risk for CRC. Both rs1558535 and rs17780195 were associated with late stages of CRC. A strong interaction was observed between rs1558535 and rs9906859 in predicting CRC risk.

## Introduction

Globally, colorectal cancer (CRC) ranks second in cancer related mortality and third in incidence^1^. By 20,230, the global burden of CRC is expected to increase by 60%in terms of new cases and deaths^2^. Traditional treatments such as radiotherapy and chemotherapy are often associated with significant adverse effects^3,4^. Although, the 5-year survival rate for CRC is approximately 64%, it drops drastically to 12% in metastatic cases^5^. In Egypt, CRC is the 7^th^ most common cancer in Egypt according to the Global Cancer Observatory^6^. Notably, Egyptian patients are typically diagnosed at late stages and face poor prognosis^7^. This underscores the urgent need for reliable and accurate prognostic molecular biomarkers.

One of the key emerging hallmarks of cancer is epigenetic dysregulation,, which is often influenced by environmental factors, inflammation, and cellular stress^8–10^. Among (epi) genetic mechanisms, non-coding RNAs (ncRNAs) have been identified as important molecular biomarkers implicated in various diseases, including multiple types of cancer^11–15^.

Long non-coding RNAs (lncRNAs), which are transcripts longer than 200 nucleotides that do not encode known proteins, play a significant role in tumorigenesis by regulating gene expression, protein synthesis, and epigenetic modifications^16,17^. Recent studies revealed that several lncRNAs were aberrantly expressed in CRC patients and were considered to be an indicator of a poor prognosis including lncRNA nicotinamide nucleotide transhydrogenase antisense RNA 1; NNT-AS1^18^, nicotinamide phosphoribosyltransferase antisense RNA 1; NAMPT-AS1^19^.

Long intergenic noncoding RNA 00,511 (LINC00511) known as onco-lncRNA-12 is a 2265 bp ncRNA that maps to chromosome 17q24.3. LINC00511has been shown to exert oncogenic functions in glioma^20^, lung cancer^21^, cervical cancer^22^, gastric cancer^23^, and CRC^24,25^. Mechanistically, LINC00511 may function as competitive endogenous RNA (ceRNA), contributing to the induction and progression of various cancers^20,22,23^ including breast cancer^26,27^. We selected LINC00511 as the focus of our study based on prior filtration and discussion processes. While genetic variations in LINC00511 have been recently detected in Chinese breast cancer patients^28^. The role of LINC00511 single nucleotide polymorphisms (SNPs) in CRC remains unexplored. Specifically, no studies to date have examined the association between LINC00511 SNPs and CRC susceptibility or related risk factors.

Therefore, to better understand the association between LINC00511 SNPs and CRC pathogenesis and severity, this study aims to quantify relevant epigenetic variations. This study aims to examine whether the genotype distribution of the tested LINC00511 SNP alleles is associated with CRC risk and/or patient outcomes by comparing colorectal cancer patients with cancer-free controls. Genetic and epigenetic variations often co-occur in specific combinations of SNPs known as haplotypes, which, when inherited together, can influence the expression of both coding and non-coding genes, including lncRNAs^29,30^. These expression changes may, in turn, impact the progression and clinical course of CRC—a hypothesis that this study seeks to explore.

### Objectives

The objectives of this study are fourfold. First, to evaluate whether LINC00511 SNP(s) variants influence CRC susceptibility by conducting a case-controlled study involving Egyptian CRC patients and cancer-free controls. This analysis will also explore whether these SNPs are associated with clinicopathological characteristics or tumor subtypes, providing a basis for precision diagnosis. Second, a logistic regression analysis will be utilized to assess the associations between LINC00511 SNPs (rs17780195 or rs9906859, and rs1558535) and CRC susceptibility or CRC risk while adjusting for potential confounders such as age, BMI, and family history.

Third, the study will investigate whether these SNPs can predict CRC prognosis by calculating odds ratios (ORs) and 95% confidence intervals (CIs) under established genetic models.

Fourth, in silico analyses using haplotype and survival association databases will be conducted to evaluate the potential role of LINC00511 SNPs in CRC outcomes. Collectively, the findings from this study are expected to inform future precision oncology strategies, including the development of targeted therapies against downstream signaling pathways regulated by LINC00511, thereby enhancing both diagnostic and therapeutic precision in CRC management.

## Subjects

### Sample size and power study

Based on the previous study by *Chong *et al.^28^, the sample size for the current study was calculated using the reported prevalence of the GG genotype of the rs17780195 SNP among breast cancer patients and healthy controls. The calculation was performed based on comparing genotype prevalence, the odds ratio (OR), between CRC cases and cancer-free controls using a **Fisher’s Exact test**, with a two-sided α-error level set at 0.05, a power of 80%, and a case-to-control ratio of 1:1. According to the referenced study, the prevalence of the GG genotype among healthy individuals was approximately **64.9%**, and the odds ratio (OR) for the association with breast cancer was approximately **0.398**. Using these parameters, including a two-sided 95% confidence level and a case-to-control ratio of 1:1, the **minimum required sample size** was estimated to be **85 participants per group** (i.e., 85 cases and 85 controls) to achieve adequate statistical power (80%) to detect a significant association, assuming the null hypothesis that the genotype distribution is equal between groups. The **Type I error probability (α)** for this hypothesis test was set at 0.05. Sample size estimation was performed using the **Power and Sample Size online calculator** for genetic association studies (G*Power), accessed in October 2021.https://csg.sph.umich.edu/abecasis/gas_power_calculator/^31^.

### Ethical Approval and Consent to Participate

All clinical and pathological data were collected using structured questionnaires. The study was approved by the Research Ethical Committee of the Faculty of Pharmacy, Ain Shams University (Approval No: 157, Date: 17 January 2023). The study was carried out in adherence to the Declaration of Helsinki Guidelines (World Medical Association Declaration of Helsinki: Ethical principles for medical research involving human subjects, 2013)^32^. Written informed consent was obtained from all participants—both CRC patients and healthy controls—after they were fully informed about the study objectives and procedures.

### Study design

Case-controlled, single-center, retrospective study.

### Study clinical trial registration

ClinicalTrials.gov Identifier: NCT06534242.

### Study participants

A total of 200 CRC patients were recruited from Mansoura University Hospitals, Mansoura, Egypt. For the control group, 200 age- and sex-matched apparently healthy individuals were randomly selected using frequency matching (±2 years). Control subjects had no history of chronic illness, were not on any medications, and had normal kidney and liver function tests. Additionally, they showed no clinical or laboratory evidence of CRC. Controls were recruited during routine health examinations—either for themselves or while accompanying relatives—or through the Chronic Diseases Screening National Presidential Program.

#### Patients’ criteria

Inclusion criteria required participants in the study to be adult with newly diagnosed CRC confirmed by pathological examination. Diagnosis was based on clinical presentation—including symptoms such as constipation, rectal bleeding, and significant weight loss—and confirmed through colonoscopy, abdominal imaging, and histopathological analysis. Only patients with CRC of no specific histological subtype were included. Exclusion criteria included any history of blood disorders, Hepatitis B or C infection, HIV, schistosomiasis, thyroid dysfunction, alcohol intake, diabetes mellitus, cardiovascular disease, or other inflammatory conditions. Patients who had received chemotherapy or radiotherapy, had undergone gastrointestinal surgical operations, or were diagnosed with any cancer other than CRC were also excluded from the study.

#### CRC patients’ clinical and pathological features

For all CRC participants, full family history of cancer and records of any prior surgical procedures were collected. Clinical staging and histological grading were determined based on colonoscopy findings, abdominal radiographic imaging, pathological analysis, and clinical judgment, following the American Joint Committee on Cancer (AJCC) 2010 criteria^33^. Tumor staging was categorized according to the TNM classification system^33,34^, as follows: stage 0 (carcinoma in situ) refers to early lesions confined to the mucosa; stage I includes small, localized tumors considered early-stage and potentially curable; stage II encompasses larger primary tumors that may extend locally but without lymph node involvement; stage III indicates regional lymph node metastasis; and stage IV denotes the presence of distant metastasis Histological grading was classified as low-grade (grade I–II), representing well or moderately differentiated tumors, or high-grade (grade III–IV), which includes poorly differentiated, undifferentiated, adenocarcinoma, or mucinous carcinoma.. Additional clinical data such as age, weight, and height were retrieved from patient records and used to calculate Body Mass Index (BMI) using the NIH BMI calculator

https://www.nhlbi.nih.gov/health/educational/lose_wt/BMI/bmicalc.htm. Participants with a BMI ≥ 25 kg/m^2^ were considered overweight or obese. Laboratory findings, including complete blood count (CBC) and classical tumor markers such as carcinoembryonic antigen (CEA) and cancer antigen 19.9 (CA19.9), were also recorded from medical files and tabulated for analysis.

## Methods

### In silico analysis

#### In silico LINC00511 gene database(s) analysis

Gene card identification was used for the identification of *LINC00511* gene and its mRNA expression in different human tissues using https://www.genecards.org/ databases (Accessed on April, 2022)^35^. *LINC00511* gene expression was obtained from RNA-seq data unit TPM, from 53 human tissue samples from the Genotype-Tissue Expression (GTEx) Project from Expression Atlas Gene expression across species and biological conditions https://www.ebi.ac.uk/gxa/home^36^ (Accessed Jan. 3^rd^, 2022).

#### *LINC00511* SNPs selection

Based on the previous study by *Chong *et al. which investigated several LINC00511 SNPs in breast cancer^28^, we screened for a SNP with a minor allele frequency (MAF) above 0.05 (≥ 5%). MAF data were obtained from the International Genome Sample Resource (IGSR) Supporting open human variation data https://www.internationalgenome.org/ in 1000 genomes data https://www.ensembl.org/ 1000GENOMES:phase 3^37^. The information on the selected Reference SNP (rs) obtained from https://www.ncbi.nlm.nih.gov/snp/ and RefSNP report^38^.

#### In silico linc00511 snps haplotype association data analysis

. EnsembI release 108—Oct 2022 © EMBL-EBI EMBL’s European Bioinformatics Institute EMBL-EBI was used to access human genomic data and annotation via https://www.ensembl.org. Pairwise linkage disequilibrium (LD) metrics—including heatmap-based pairwise correlation coefficients (R^2^) and standardized linkage disequilibrium values (D′)—were obtained for the three tested LINC00511 SNP variants, The analysis was conducted using data from the **1000 Genomes Project Phase 3**, specifically the **YRI (Yoruba in Ibadan, Nigeria) population**, to assess the genetic correlation and potential haplotype structure among the selected SNPs. Direct access to variant-level data and visualization tools was provided through the Ensembl variation browser at http://www.ensembl.org/Homo_sapiens/Info/Index?db=core;r=17:72618050-72638049;v=rs17780195;vdb=variation;vf=106739215

#### LINC00511 gene differential expression and snps survival platform analysis

Pan-Cancer Survival Analysis for *LINC00511* gene was conducted across 32 different types of Cancers, comprising a total of 10,882 RNA-seq samples. Among these, expression data for colon adenocarcinoma were specifically examined. The RNA-seq expression data of colon adenocarcinoma were downloaded from The Cancer Genome Atlas Program; TCGA project https://cancergenome.nih.gov/ via Harmonized Cancer Datasets; Genomic Data Commons Data Portal https://portal.gdc.cancer.gov from the National Cancer Institute GDC Data Portal^39,40^. LINC00511 expression values were log-transformed using the formula log2(FPKM + 0.01) to normalize the data. For pan-cancer comparative analysis, expression profiles were retrieved from ENCORI – The Encyclopedia of RNA Interactomes https://starbase.sysu.edu.cn/panCancer.php, which integrates ~ 10,000 RNA-seq and ~ 9,900 miRNA-seq samples from the TCGA project^41^.. This analysis enabled cross-cancer comparisons and evaluation of LINC00511’s expression pattern and prognostic significance, including in colorectal cancer.

### Blood samples

Four mls of peripheral blood were withdrawn from CRC patients (collected at the time of diagnosis for those who met the inclusion criteria, and signed the IC, and were rejected for those who met the exclusion criteria) and controls, under strict sterile conditions for molecular testing, on EDTA anticoagulant vacutainers and stored at −80º C, until DNA extraction and biochemical assessment at the Advanced Biochemistry Research Lab, Faculty of Pharmacy, Ain Shams University (Research Setting), Abassia, Cairo, Egypt.

#### DNA Extraction

DNA was **extracted** from 200 μL whole blood using a QIAmp DNA Blood Mini extraction kit (Qiagen, Valenica, CA) according to the manufacturer’s instructions.

#### DNA Quantification

The yield was quantified, and its purity was measured by Platinum-colored DS11 Spectrophotometer (DeNovix Inc, USA) and stored at −80°C, until assessment.

#### SNPs Genotyping

Was performed using predesigned TaqMan® SNP genotyping assays for rs17780195, rs9906859, and rs1558535 (catalog no: 4351379, Thermo Fisher Scientific, USA) and TaqMan genotyping Master Mix (Catalog no: 4371353, Thermo Fisher Scientific, USA). For each sample, 20 ng of DNA template genotyped using 10 μL (2×) TaqMan® genotyping Master Mix, 0.5 μL (40×) TaqMan® SNP genotyping assay, and continued by DNAse/RNAse-Free water (Gibco, Life Technologies, USA) to a total volume of 20 μL reaction using default settings for genotyping with the appropriate negative control. The real-time polymerase chain reaction (RT-PCR) was performed on StepOne Plus Thermal Cycler (Applied Biosystems, USA).

### Data statistical-analysis

The statistical analysis was performed using R software (version 4.2.0). First, for each studied SNP, genotype frequencies were checked to be in concordance with the Hardy–Weinberg Equilibrium (HWE) using Pearson’s χ^2^ Chi-squared tests. Qualitative variables were expressed as number and percentage while quantitative data were expressed as median and interquartile range (IQR). Mann–Whitney’s W Test and the *χ*^2^ test were used to compare quantitative and qualitative variables between the CRC and control groups, respectively. After adjusting for demographic factors (age, sex, BMI, family history of cancer in first degree relatives), an unconditional logistic regression applied to explore the independent risk factors for CRC among the examined LINC00511 target SNPs using the co-dominant, dominant, over-dominant, and recessive genotypic models, then being compared using measures of model fit and prediction (the Akaike Information Criterion (AIC), Bayesian Information Criterion (BIC), Deviance Information Criterion (DIC), Pseudo R^2^ (McFadden’s, Cox and Snell’s, and Nagelkerke’s), and the area-under-receiver operating characteristics (AUC) curve. The additive/co-dominant model was superior to all models based on these criteria. Sensitivity (SN), specificity (SP), positive predictive value (PPV), and negative predicted value (NPV) were reported for the model with the best fit and predictive power (not for mere disease diagnosis). The optimal cut-offs for age, CA19-9, and CEA categorization of LINC00511 SNPs were determined using ROC analysis. SNPs genotypes were analyzed using the snpReady library in R^42^. Missing genotypes were imputed using Wright’s method (based on Wright’s equilibrium) and missing baseline demographic and clinical data were imputed using the predictive mean matching method implemented in R^43,44^.

Multiple logistic regression analysis was done to examine the association between each SNP alleles, genotypes, haplotypes, and CRC prevalence, stage, and grade, while adjusting for baseline covariates (age, BMI, and additional risk factors for tumor stage and tumor grade, family history, tumor site, history of inflammatory bowel disease (IBD)), classical tumor markers (CEA and CA19.9), and the presence or absence of vascular infiltration.

Firth’s logistic regression was implemented in the case of quasi-separation in one or more variables. Haploview software version 2.0 was used to calculate *r*^*2*^ and D’ as the measurements of linkage disequilibrium extent between pairwise SNP combinations blocks of different genotypes were determined using the SHEsis software http://analysis.bio-x.cn/myAnalysis.php^45^. A stratified analysis and the SHEsis plus online software http://shesisplus.bio-x.cn/SHEsis.html applied to further evaluate the association between LINC00511 SNPs and CRC susceptibility as well as haplotypes frequency as a measurement of genetic distribution was directly calculated in CRC and healthy control groups. Further, Epistasis was analyzed by Multifactor Dimensionality Reduction (MDR) package software in R https://ritchielab.org/software/mdr-download for carrying out SNP-SNP or gene–gene and SNP-Environment or gene-environment interaction analysis, applied to evaluate the interactive role of genetic and demographic factors (false Discovery rate was controlled by adjusting the significance level using Benjamini and Hochberg, Benjamini and Yekutieli, Holm step-down, Sidak step-down, and Sidak single-step *p*-value adjustment procedures). When comparing the predictive ability of the models using the pseudo-R-squared measures and the SN, SP, PPV, and NPV by ROC curve, in addition to the MDR part, aids for LINC00511 SNPs rs prediction. Prediction method LD values were calculated by a pairwise estimation between LINC00511 SNPs genotyped in the same sample and within a given window. An established method was used to estimate the maximum likelihood of the proportion that each possible haplotype contributed to the double heterozygote. To confirm differences between obtained results in the patient group were not by chance, a Bonferroni calculation was applied to the data^46^.

Finally, we analyzed the overall survival (OS) for 1–3 years in the CRC patients (n = 200) with recording the date of death or last contact with the Clinician as the follow-up end point. Median follow-up time for the patients was 1 year. For disease-free survival (DFS), as the event free survival (EFS), in patients with non-metastatic CRC at the time of diagnosis, date of relapse or last contact with the Clinician was the follow-up end point. Median follow-up time was 1 years. The non-parametric Kaplan–Meier method was done for OS curves and EFS. The survival probability calculator generates the Kaplan–Meier curve with 95% CI, using log-rank test using the Chi square distribution, for comparison of more than of two groups.

The relative risk of disease relapse was estimated as hazard ratio (HR). Univariable survival analyses were done for gender, MBI, tumor site, family history of cancer, lymph node involvement, TNM stage and tumor grades separately, for the CRC patients’ group as well as for LINC00511 SNPs genotypes. A two-sided value of *P* < 0.05 was deemed as a sign of statistical significance.

## Results

### LINC00511 gene In silico databases analysis

**Figure. 1:**
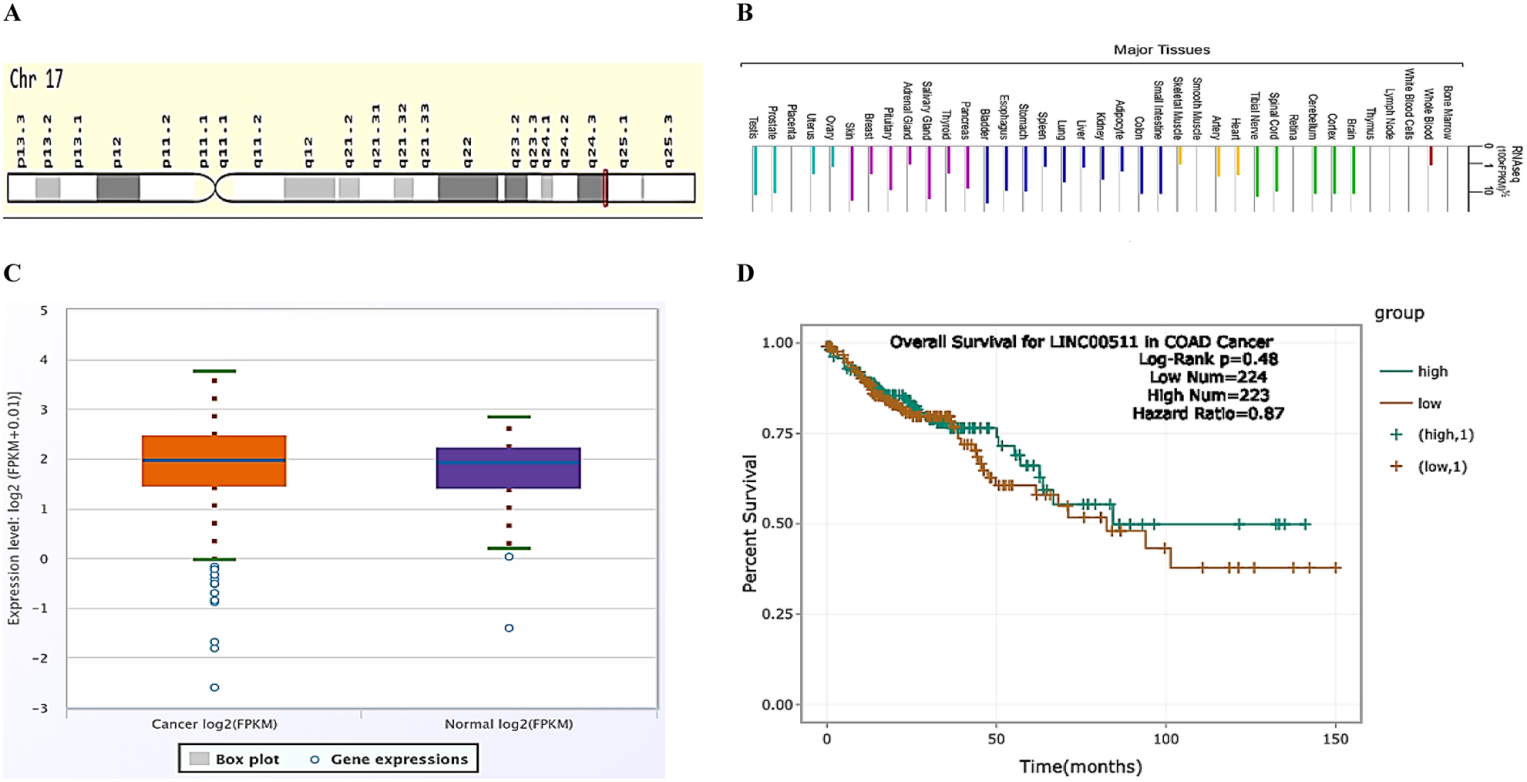
**A**) *LINC00511* genomic location: bands according to Ensembl https://www.genecards.org/cgi-bin/carddisp.pl?gene=LINC00511 locations according to GeneLoc. Accessed on April, 2022 and **B**) *LINC00511* gene mRNA expression in normal human tissues from RNAseq https://www.genecards.org/cgi-bi n/carddisp.pl?gene = LINC00511#function showing sigmoid and transverse colon (Accessed on April, 2022). Accessed Jan. 3^rd^, 2022. **C**) *LINC00511* gene differential expression in 471 colon adenocarcinoma vs 41 normal samples https://rnasysu.com/encori/panGeneDiffExp.php#COAD Data source is ENCORI project. **D**) *LINC00511* gene overall survival plot from Pan-Cancer Survival Analysis for Colon Adenocarcinoma by ENCORI_ The Encyclopedia of RNA Interactomes https://starbase.sysu.edu.cn/panGeneSurvivalExp.php# Accessed on Jan. 3^rd^, 2023).

We used the HUGO Gene Nomenclature Committee (HGNC), supported by the National Human Genome Research Institute (NHGRI) https://www.genenames.org/data/gene-symbol-report/#!/hgnc_id/HGNC:43564, to provide the Symbol report for *LINC00511*. *LINC00511* is an oncogene lncRNA located on chromosome 17, its cytogenetic band is 17q24.3 identified by HGNC and Entrez Gene or Ensembl algorithms. *LINC00511* gene card (GC) id is GC17M072323 and its length is 2,265 bp being made up of 5 exons https://www.genecards.org/cgi-bin/carddisp.pl?gene=LINC00511&keywords=LINC00511 (Accessed on April, 2022) (Fig. 1A). Using gene cards algorithm RNAseq https://www.genecards.org/cgi-bin/carddisp.pl?gene=LINC00511#function, *LINC00511* mRNA expression was obvious in sigmoid and transverse colon (Accessed on April, 2022) (Fig. 1B).* LINC00511* differential gene expression box plot (Fig. 1C) from the ENCORI project in 471 colon adenocarcinoma patient’s vs 41 normal samples. https://rnasysu.com/encori/panGeneDiffExp.php#COAD Pan-Cancer Survival Analysis of Genes across 32 types of Cancers across 447 samples and HR 0.87 was non-significant (p = 0.48) for LINC00511 gene. https://rnasysu.com/encori/panGeneSurvivalExp.php#COAD (Accessed on Jan. 3rd, 2023) (Figure 1D).

#### Selected SNPs criteria

Based on the previous study by *Chong *et al. 2020 that investigated several LINC00511 SNPs, and following screening for SNPs with a MAF above 0.05, three SNPs located within the LINC00511 gene were selected for analysis: rs1558535 (MAF = 0.36), rs17780195 (MAF = 0.18), and rs9906859 (MAF = 0.47). These SNPs were chosen by our research group to evaluate their potential association with colorectal cancer (CRC). Detailed information about the selected SNPs is provided in Table 1. The rs of selected SNPs report obtained from https://www.ncbi.nlm.nih.gov/snp/ and RefSNP report. MAF obtained from the International Genome Sample Resource (IGSR) Supporting open human variation data https://www.internationalgenome.org/ in 1000 genomes data https://www.ensembl.org/ as reported previously. The pathogenic allele in the rs1558535 is T, and for rs17780195 is G, however, it is C for rs9906859.

**Table 1:** LINC00511 SNPs rs9906859, rs1558535, and rs17780195 allele, chromosome, functional consequence, and MAF info report.

	**LINC00511 SNPs**		
	**rs1558535**	**rs17780195**	**rs9906859**
**Allele**	**[A > T]**	**[A > G]**	**[T > A, C]**
**Allele Flanks**	AACAAAACAAAACAAAACAAAACACAAAGACAGTCACAGCAACTACTGAGACGGGAAAGCTATCATGGCATGGTGTGCATGCCTGTATTTTAAAACAGAC	CTCCTCATCCGCCACAACAGGAGACAAAACTGAACCAGGGAGAGTCCAGATGTGGAGACTGCATAGGGCAGGGGCAGCACAAGGACCAGAAGACAGGGGT	GACAGGGGTAAAAAAGAAATGTCTACATGCCCCTTCACCTAGTTTGCTCTTCAAATGTTAGAGACCAACTTACAATCCACCCATGAGAACGGTCTGGATT
	**[A/T]**	**[A/G]**	**[T/A,C]**
	GATACTTACAGACTATCTTCCGGCTAAACATTTTCCTAAGTTTTGGAAGAAGTCTCAATAAATTGGAACAAGTCTCAATTTTCCTGCTCAGAATTCTCTG	AAAAAGAAATGTCTACATGCCCCTTCACCTAGTTTGCTCTTCAAATGTTAGAGACCAACTTACAATCCACCCATGAGAACGGTCTGGATTTCTCTGTGGT	CTCTGTGGTAAGATAGATCATCCCAAGAGAAAACCCTAAACATAGTGATATTTAAGAGTCTCCAGTGAAAAGTGGAGTCTCTCTCTAAGTGCCCTACAGC
**FAM/VIC**	**T/A**	**G/A**	**T/C**
**Chromosome**	17:72,612,450 (GRCh38) 17:70,608,589 (GRCh37)	17:72,628,050 (GRCh38) 17:70,624,189 (GRCh37)	17:72,628,141 (GRCh38) 17:70,624,280 (GRCh37)
**Canonical SPDI**	NC_000017.11:72,612,449:A:T	NC_000017.11:72,628,049:A:G	NC_000017.11:72,628,140:T:A, NC_000017.11:72,628,140:T:C
**Functional Consequence**	Intron variant	Upstream transcript variant, 2KB upstream variant, intron variant	2KB upstream variant, upstream transcript variant, intron variant
**MAF**			
1000Genomes	T = 0.360823/1807	G = 0.178315/893(G)	T = 0.472045/2364

For all the variant type is SNV, intron variants, being validated by frequency, by alfa, by cluster. Reference SNP (rs) Report https://www.ncbi.nlm.nih.gov/snp/ and Ensembl genome browser 110, [LINC00511: Long Intergenic Non-Coding RNA 00,511, MAF: Minor Allele Frequency, SNP: Single Nucleotide Polymorphism, SPD: Sequence Position Deletion Insertion.] Via the SNPinfo web server, LINC00511 SNP rs9906859 info in DNA sequence https://manticore.niehs.nih.gov/cgi-bin/snpinfo/snpinfo1.cgi#t168135875 neighbor rs is rs17780195.

In the current study, the three LINC00511 SNPs were in strong linkage disequilibrium (LD) and were moderately correlated as appears in Table 2.

**Table 2:** Linkage disequilibrium and correlation between LINC00511 studied SNPs.

**LINC00511 SNPs**	**Statistics**	**LINC00511 SNPs**	
		**rs9906859**	**rs17780195**
**rs1558535**	D	0.16	−0.07
	D’	0.75	0.66
	*R*^*2*^	0.66	−0.35
	χ^2^	344	95.8
	***P***	< 0.001*	< 0.001*
**LINC00511 SNPs**	**Statistics**	**rs9906859**	
**rs17780195**	D	−0.08	
	D’	0.83	
	*R*^*2*^	−0.38	
	χ^2^	118	
	***P***	< 0.001*	

[D: linkage disequilibrium, D’: Normalized linkage disequilibrium, LINC00511: Long Intergenic Non-Coding RNA 00,511, *R*^*2*^: correlation coefficient, χ^2^: Pearson’s Chi-squared test.].

### LINC00511 SNPs Allele/Genotype frequencies

LINC00511 SNPs rs1558535, rs17780195, and rs9906859 were checked for agreeing with the HWE using Pearson’s χ 2 Chi-squared tests. All LINC00511 studied SNPs had call rates more than 0.95 and MAF greater than 0.05, in all the investigated groups. Imputed genotypes comprised 1.9% of all data. There was a significant deviation from the HWE in LINC00511 SNP rs1558535 in controls (χ2 = 10.5, P=0.001), and for LINC00511 SNP rs9906859 in controls (P < 0.001) as well as for all the study participants (n=400) (P=0.047) (Table 3).

**Table 3:** LINC00511 SNPs rs1558535, rs17780195, and rs9906859 frequencies attributes and HWE testing in the studied groups.

**LINC00511 SNPs**	**Group, N**								
	**CRC, 200**			**Controls, 200**			**All participants, 400**		
	**MAF**	**H**_**e**_	***p***	**MAF**	**H**_**e**_	***P***	**MAF**	**H**_**e**_	***p***
**rs1558535 A > T**	0.47 (A)	0.5	NS	0.48 (A)	0.5	**0.001**	0.48 (A)	0.5	NS
**rs17780195 A > G**	0.22 (G)	0.35	NS	0.24 (G)	0.36	NS	0.23 (G)	0.36	NS
**rs9906859 T > C**	0.39 (T)	0.48	NS	0.43 (T)	0.49	**0.001**	0.41 (T)	0.48	**0.04**

Pearson’s χ^2^ Chi-squared test was used, statical significance at *p* < 0.05. [H_e_: Expected Heterozygosity, CRC: Colorectal cancer, LINC00511: Long intergenic non-coding RNA 00,511, MAF: Minor allele frequency, SNP: Single nucleotide polymorphism, NS: Non-significant.].

### Subjects demographic basic data and snps results

The median age of the whole study group was 45 years, range (20—78 years). Patients were significantly older than the healthy controls with median age 53 vs. 38 years, (*P* < 0.001). A significantly higher proportion of CRC patients were overweight or obese (BMI ≥ 25 kg/m^2^) compared to healthy controls (79.0% vs. 64.5%, P < 0.01), as shown in Table 4. In addition, rs1558535 AT and variant was more frequent in CRC patients than controls (55.5% vs. 37.0%, P < 0.01). Also, rs9906859 TC variant was higher in CRC cases than controls (52.0% vs. 35.5%, P < 0.01). CRC patients carrying wild genotype of both rs1558535 and rs9906859 were much less than controls carrying such genotype (19.0% and 13.0% vs. 30.6% and 25.0%, respectively).

**Table 4:** Baseline age and BMI as well as LINC00511 SNPs rs1558535, rs17780195, rs9906859 genotypes in CRC patients (n = 200) vs control group (n = 200).

**Variable (unit)**	**Group, N**		
	**CRC, 200**	**Controls, 200**	**Statistics, *P***
**Age**^**#**^** (years)**	53 (44—61)	38 (31.4—47.0)	W = 31,779, < **0.01**^1^
**Age** > 40	173 (86)	81 (40)	< 0.001*
**BMI** ≥ 25 (kg/m^2^)	158 (79.0)	129 (64.5)	χ^2^_1_ = 10.4, < **0.01**^2^
**LINC00511 SNPs**			
**rs1558535 A > T**			χ^2^_2_ = 14.2, < **0.01**^2^
AA	39 (19.5)	61 (30.5)	
AT	111 (55.5)	74 (37.0)	
TT	50 (25.0)	65 (32.5)	
**rs17780195 A > G**			χ^2^_2_ = 3.02, NS
AA	116 (58.0)	117 (58.5)	
AG	78 (39.0)	70 (35.0)	
GG	6 (3.0)	13 (6.5)	
**rs9906859 T > C**			χ^2^_2_ = 14.4, < **0.01**^2^
TT	26 (13.0)	50 (25.0)	
CT	104 (52.0)	71 (35.5)	
CC	70 (35.0)	79 (39.5)	

All data are N (%), ^#^ Statistics are expressed as median (IQR), ^1^Mann-Whitney’s W Test. ^2^Pearson’s χ^2^ Chi-Squared Test. Age, BMI optimal cut-offs for disease prediction were determined using ROC curve analysis. [BMI: Body-Mass Index, LINC00511: Long Intergenic Non-Coding RNA 00,511, NS: Non-significant, SNP: Single Nucleotide Polymorphism.].

### Association tests

#### Single locus (Alleles) association tests

Comparisons between alleles proportions in CRC patients vs. healthy controls showed no difference in the proportions of the pathogenic allele in rs1558535 (53% vs. 54%), rs17780195 (23% vs. 24%), and rs9906859 (61% vs. 57%) (Table 5). Similarly, logistic regression analysis, adjusted for age and BMI category, showedno association between the allele type and CRC in rs1558535 (OR: 1.05, 95% CI: 0.77—1.43), rs17780195 (OR: 0.88, 95% CI: 0.60—1.30), and rs9906859 (OR: 1.09, 95% CI: 0.81—1.49).

**Table 5:** Association between LINC00511 SNPs rs1558535, rs17780195, and rs9906859 allele frequencies (proportion) in CRC patients (n = 200) vs. controls (n = 200).

LINC00511 SNPs	Group	Allele frequency, N (%)		OR (95% CI), *p* value
				OR^#^ (95% CI), *p* value
rs1558535 A > T		A	T	1.03 (0.78 — 1.36), NS 1.05^#^ (0.77 — 1.43), NS
	CRC	188 (47)	212 (53)	
	Control	185 (46)	215 (54)	
rs17780195 A > G		A	G	0.92 (0.66 — 1.28), NS 0.88^#^ (0.60 — 1.30), NS
	CRC	310 (77)	90 (23)	
	Control	304 (76)	96 (24)	
rs9906859 T > C		T	C	1.17 (0.88 — 1.54), NS 1.09^#^ (0.81 — 1.49), NS
	CRC	156 (39)	244 (61)	
	Control	171 (43)	229 (57)	

Pearson’s Chi-Squared Test for odds ratio calculation, ^#^Odds ratios adjusted for age (< 40 vs. > 40 yr) and BMI (≤ 25 kg/m^2^), [BMI: Body mass index, CRC: colorectal cancer, LINC00511: Long intergenic non-coding RNA 00,511, NS: Non-significant, SNP: Single nucleotide polymorphism].

#### Genotype association tests

Concerning the tumor grade and stage association to LINC00511 SNPs presented in Tables 6 and 7, respectively. First, none of the studied SNPs correlated with tumor grade (Table 6). rs1558535 AT variant correlated with more advanced stages III or IV diseases compared to earlier stages I or II (67% vs. 41%, *P* < 0.001) and a nearly fourfold increase in the odds of presenting with CRC stages III or IV (adjusted OR: 3.99, 95% CI: 1.30—13.0, *P* = 0.022). The rs17780195 AG variant correlated with more advanced stages III or IV diseases compared to earlier stages I or II (46% vs. 30%, *P* < 0.045) and a nearly threefold increase in the odds of presenting with CRC stages III or IV (adjusted OR: 2.72, 95% CI: 1.28—6.02, *P* = 0.011). On the other hand, rs9906859 did not correlate with the tumor stage (Table 7). Interestingly, data shown in Table 6 and 7 revealed that gender, family history and IBD history are associated with high tumor grades with OR 3.04, 4.02 and 125.04, respectively, at *P* < 0.05. In addition, transverse tumor site, vascular infiltration, and patients having CEA > 4 ng/mL or CA 19–9 > 40 U/mL are associated with late CRC stages.

**Table 6:** Co-dominant Model for CRC group (n = 200) tumor grade, low or high, association with demographic characteristics (age, BMI, sex, and family cancer history) and tumor clinical features (tumor site and vascular infiltration, IBD, and blood TMs) with LINC00511 SNPs rs1558535, rs17780195, and rs9906859 genotypes.

**Characteristic (Unit)**	**Grade, N (%)**		***P1***	**OR (95% CI)**	***P2***
	**low**, (n = 161)	**high**, (n = 39)			
**Age (year)**			** < 0.001**		
< 55	106/161 (66)	12/39 (31)		1.00	
> 55	55/161 (34)	27/39 (69)		2.55(0.9 — 7.17)	NS
**BMI (Kg/m**^**2**^**)**			NS		
< 25	36/161 (22)	6/39 (15)		1.00	
≥ 25	125/161 (78)	33/39 (85)		1.41(0.46 — 4.3)	NS
**Sex**			**0.002**		
Female	120/161 (75)	19/39 (49)		1.00	
Male	41/161 (25)	20/39 (51)		3.04(1.18 — 7.81)	**0.025**
**Family History**			**0.010**		
Negative	127/161 (79)	23/39 (59)		1.00	
Positive	34/161 (21)	16/39 (41)		4.02(1.45 — 11.18)	**0.009**
**Tumor site**			NS		
Colon	97/161 (60)	23/39 (59)		1.00	
Rectal	64/161 (40)	16/39 (41)		0.6 (0.24 — 1.47)	NS
**Vascular infiltration**			NS		
Absent	79/161 (49)	17/39 (44)		1.00	
Present	82/161 (51)	22/39 (56)		0.94 (0.36 — 2.46)	NS
**IBD History**			** < 0.001**		
Negative	98/161 (61)	0/39 (0)		1.00	
Positive	63/161 (39)	39/39 (100)		125.04(11.2 — 1392)	** < 0.001**
**CEA (ng/mL)**			NS		
< 9	73/161 (45)	20/39 (51)		1.00	
> 9	88/161 (55)	19/39 (49)		0.90 (0.37 — 2.2)	NS
**CA19-9 (U/mL)**			**0.025**		
< 73	120/161 (75)	22/39 (56)		1.00	
> 73	41/161 (25)	17/39 (44)		2.13 (0.76 — 6)	NS
**LINC00511 SNPs**					
**rs1558535 A > T**			NS		
AA	33/161 (20)	6/39 (15)		1.00	
AT	86/161 (53)	25/39 (64)		5.51 (0.12 — 2.15)	NS
TT	42/161 (26)	8/39 (21)		0.53 (0.08 — 3.65)	NS
**rs17780195 A > G**			NS		
AA	93/161 (58)	23/39 (59)		1.00	
AG	64/161 (40)	14/39 (36)		1.39 (0.53 — 3.68)	NS
GG	4/161 (2.5)	2/39 (5.1)		0.2 (0.02 — 2.24)	NS
**rs9906859 T > C**			NS		
TT	24/161 (15)	2/39 (5.1)		1.00	
CT	81/161 (50)	23/39 (59)		1.90 (0.23 — 15.58	NS
CC	56/161 (35)	14/39 (36)		7.43 (0.65 — 85.38)	NS

**Table 7:** Co-dominant model table for CRC group (n = 200) tumor stages association with demographic characteristics (age, BMI, sex, and family cancer history) and tumor clinical features (tumor site and vascular infiltration, IBD, and blood TMs) with LINC00511 SNPs rs1558535, rs17780195, and rs9906859 genotypes.

**Characteristic (Unit)**	**Stage, N (%)**				
	**I-II**, (n = 88)	**III-IV**, (n = 112)	***P1***	**OR (95% CI)**	***P2***
**Age (year)**			NS		
< 55	54/88 (61)	64/112 (57)		1.00	
> 55	34/88 (39)	48/112 (43)		1.10 (0.51 — 2.41)	NS
**BMI (Kg/m**^**2**^**)**			NS		
< 25	20/88 (23)	22/112 (20)		1.00	
≥ 25	68/88 (77)	90/112 (80)		1.66 (0.73 — 3.81)	NS
**Sex**			NS		
Female	67/88 (76)	72/112 (64)		1.00	
Male	21/88 (24)	40/112 (36)		1.99 (0.93 — 4.38)	NS
**Family History**			NS		
Negative	68/88 (77)	82/112 (73)		1.00	
Positive	20/88 (23)	30/112 (27)		1.63 (0.72 — 3.76)	NS
**Tumor site**			NS		
Ascending Colon	51/88 (58)	45/112 (40)		1.00	
Transverse Colon	5/88 (6)	19/112 (17)		4.31 (1.49–12.47)	**0.004**
Descending Colon	32/88 (36)	48/112 (43)		1.7 (0.93–3.1)	**0.05**
**Vascular infiltration**			** < 0.001**		
Absent	54/88 (61)	42/112 (38)		1.00	
Present	34/88 (39)	70/112 (62)		4.00 (1.89 — 8.89)	** < 0.001**
**IBD History**			NS		
Negative	40/88 (45)	58/112 (52)		1.00	
Positive	48/88 (55)	54/112 (48)		0.33 (0.14 — 0.73)	**0.008**
**CEA (ng/mL)**			** < 0.001**		
< 4	37/88 (42)	20/112 (18)		1.00	
> 4	51/88 (58)	92/112 (82)		3.55 (1.66 — 7.89)	**0.001**
**CA19-9 (U/mL)**			**0.017**		
< 40	42/88 (48)	35/112 (31)		1.00	
> 40	46/88 (52)	77/112 (69)		2.82 (1.31 — 6.32)	**0.009**
**LINC00511 SNPs**					
**rs1558535 A > T**			** < 0.001**		
AA	24/88 (27)	15/112 (13)		1.00	
AT	36/88 (41)	75/112 (67)		3.99 (1.30 — 13.0)	**0.018**
TT	28/88 (32)	22/112 (20)		0.51 (0.09 — 2.66)	NS
**rs17780195 A > G**			**0.032**		
AA	58/88 (66)	58/112 (52)		1.00	
AG	26/88 (30)	52/112 (46)		2.72 (1.28 — 6.02)	**0.011**
GG	4/88 (4.5)	2/112 (1.8)		1.24 (0.12 — 11.0)	NS
**rs9906859 T > C**			NS		
TT	16/88 (18)	10/112 (8.9)		1.00	
CT	42/88 (48)	62/112 (55)		1.52 (0.41 — 5.88)	NS
CC	30/88 (34)	40/112 (36)		2.66 (0.50 — 15.2)	NS

***P1****, Pearson’s Chi-Squared Test.* Odds ratios are obtained by Firth’s logistic regression analysis. Odds ratios are adjusted for age, BMI, sex, family history tumor site, vascular infiltration, IBD history, CEA, and CA19-9 at baseline. ***P2***; *P* values for odds ratios and corrected for multiple testing using Benjamini and Hochberg’s method. Age, CEA, and CA19-9 optimal cut-offs for grade prediction were determined using ROC curve analysis.

[BMI: Body mass index, CA-19–9: Carbohydrate antigen 19–9, CEA: Carcinoembryonic antigen, IBD: Inflammatory bowel disease, LINC00511: Long intergenic non-coding RNA 50,011, NS: non-significant, OR: Odds ratio, CI: Confidence interval.]

***P1****, Pearson’s Chi-Squared Test.* Odds ratios are obtained by Firth’s logistic regression analysis. Odds ratios are adjusted for age, BMI, sex, family history tumor site, vascular infiltration, IBD history, CEA, and CA19-9 at baseline. ***P2***; *P* values for odds ratios and corrected for multiple testing using Benjamini and Hochberg’s method. Age, CEA, and CA19-9 optimal cut-offs for grade prediction were determined using ROC curve analysis.

[BMI: Body mass index, CA-19–9: Carbohydrate antigen 19–9, CEA: Carcinoembryonic antigen, IBD: Inflammatory bowel disease, LINC00511: Long intergenic non-coding RNA 50,011, NS: non-significant, OR: Odds ratio, CI: Confidence interval.]

To explore the independent risk factors for CRC among the examined LINC00511 SNPs, unconditional logistic regression was applied, compared model fit and prediction using the co-dominant genotypic model, WSD significantly better than the null model. The co-dominant, and over-dominant models showed comparable adequacy of fit (DIC: 446 vs. 449, AIC: 464 vs. 461, BIC: 500 vs. 485), and predictive ability demonstrated by comparable AUCs (0.786 vs 0.784) and pseudo R^2^ values (McFadden’s: 0.196 vs. 0.190, Cox and Snell’s: 0.238 vs. 0.232, Nagelkerke’s: 0.317 vs. 0.309) (Table 1S). In the co-dominant model, rs1558535 genotypes were independently correlated with CRC; The frequency of the wild-type AA variant was greater in controls compared to cases (30% vs. 20%, *P* = 0.020) and the frequency of the heterozygous AT variant was greater in cases than controls (56% vs. 39%, *P* < 0.001). However, when adjusted for age and BMI, the association between rs1558535 variants and CRC was insignificant. rs9906859 independently correlated with CRC; the frequency of the wild-type TT variant was greater in controls compared to cases (26% vs. 13%, *P* = 0.002) and the frequency of the heterozygous TC variant was greater in cases than controls (52% vs. 34%, *P* = 0.001). In addition, when adjusted for age and BMI, the heterozygous TC variant was associated with a more than threefold increase in the odds of CRC (OR: 3.54, 95% CI: 1.58–8.13, *P* = 0.002). Moreover, both dominant and over-dominant models showed association between rs9906859 and increased risk for CRC (OR: 3.04, 95% CI: 1.43–6.64, *P* = 0.004 and OR: 2.57, 95% CI: 1.49–4.52, *P* = 0.001, respectively). For rs17780195, there were no associations between any of its variants and CRC (*P* = 0.221) (Table 2S).

#### Haplotype analysis

A significant correlation between LINC00511 SNPs rs1558535, rs17780195, and rs9906859 haplotype and CRC was observed (*P* < 0.001). Post-hoc analysis revealed that the ‘TAC’ haplotype—comprising the mutant allele for rs1558535, wild-type for rs17780195, and mutant for rs9906859—was associated with a modestly increased risk of CRC (observed in 31.5% of cases vs. 24.0% of controls; P = 0.020). This haplotype conferred an approximately 1.5-fold increased risk of CRC (OR: 1.46; 95% CI: 1.07–1.99), and the association remained statistically significant after adjustment for false discovery rate.. On the other hand, the ‘TAT’ mutant-wild-wild haplotype conferred lower risk of CRC (Cases: 1.7%, Controls: 8%, *P* < 0.001) and fivefold lower odds of CRC (OR: 0.20, 95% CI: 0.09–0.47). Adjusting for false detection rate did not impact the association between the ‘TAT” haplotype and CRC (Table 8).

**Table 8:** Haplotype analysis of LINC00511 SNPs rs1558535, rs17780195, and rs9906859 in CRC (n = 200).

**LINC00511 SNPs**	**Group, N (%)**					**FDR adjustment**		
Haplotype*	**CRC, 200**	**Control, 200**	***X***^**2**^	***P***	**OR (95% CI)**	**Holm**	**BH**	**BY**
TAC higher risk	63 (31.5%)	48 (24%)	5.61	0.02*	1.46 (1.07—1.99)	0.049*	0.03*	NS
TGC	39 (19.5%)	42 (21%)	0.38	NS	0.90 (0.63—1.27)	0.749	0.54	1.00
AAT	74 (37.0%)	68 (34%)	0.79	NS	1.14 (0.85—1.52)	0.749	0.43	1.00
AAC	15 (7.5%)	20 (10%)	1.87	NS	0.71 (0.43—1.16)	0.514	0.23	0.62
TAT lower risk	3 (1.5%)	16 (8%)	16.8	< 0.001*	0.20 (0.09—0.47)	< 0.001*		

Global χ^2^ is 22.6, *P* < 0.001. Using the Marchov model. * Haplotypes with frequencies less than 0.03 were ignored. Global χ^2^ = 22.6, *P* < 0.001. *Alleles in each haplotype are sorted from left to right as rs1558535 A/T, rs17780195 A/G, and rs9906859 T/C. Haplotype phasing is performed by SHEsisPlus, based on partition-ligation-combination- subdivision expectation maximization algorithm (PLCSEM). [BH: Adjusted *p*-values per the Benjamini and Hochberg procedure, BY: Adjusted *p*-values per the Benjamini and Yekutieli procedure, FDR: False-Detection Rate, Holm: Holm step-down adjusted p-values, LINC00511: Long intergenic non-coding RNA 50,011, SNP: Single nucleotide polymorphism, NS: Non-significant, OR: Odds ratio, CI: Confidence interval.].

### Gene interaction analysis (GIA)/Epistasis analysis based on Shannon’s entropy

**Fig. 2:**
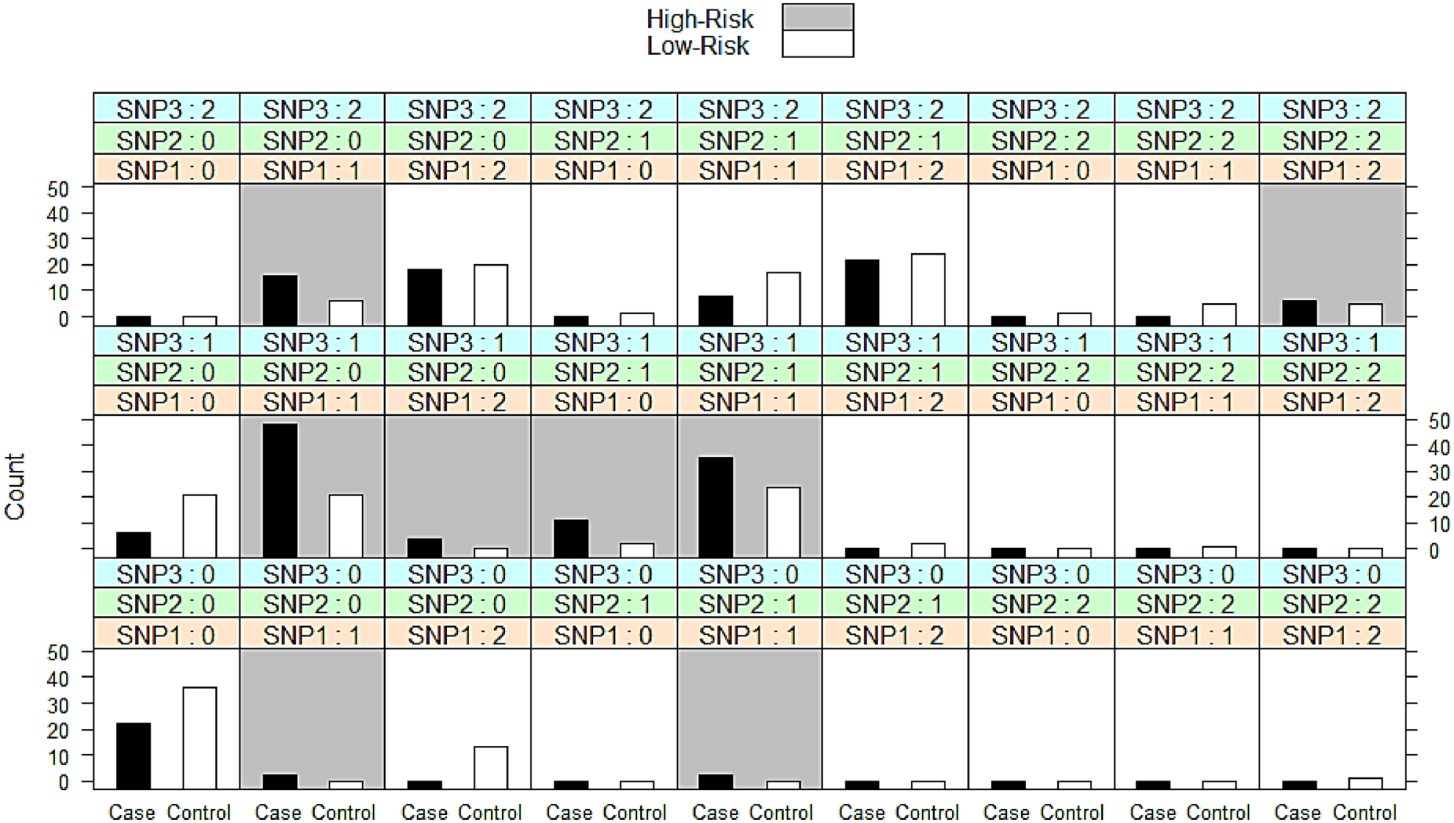
Multifactor dimensionality reduction (MDR) model fit with a three-way split for LINC00511 SNPs for CRC cases (n = 200) and controls (n = 200). Epistasis analysis is based on Shannon’s Entropy flowing chi square distribution. [SNP1 is rs1558535, SNP2 is rs17780195, and SNP3 is rs9906859. Genotypes are coded ‘0’ for the wild-type, ‘1’ for heterozygous, and ‘2’ for homozygous. Grey bars denote high-risk and the white bars for low-risk.].

To identify and visualize the specific multi-locus genotype combinations involving the 3 studied SNPs that are associated with either high or low-risk of CRC, Multi-Factor Dimensionality Reduction (MDR) interaction plot was used (Fig. 2). The MDR analysis showed that individuals who are all-heterozygous or all-homozygous for the alternate allele in all studied loci are at high risk of CRC. Finally, individuals who are heterozygote and homozygote or homozygote and heterozygote for rs1558535 and rs9906859, respectively (i.e. TT/AG/TC or AT/AG/CC) are at high risk of CRC as well, suggesting that the interaction between rs1558535 and rs9906859 is more predictive of CRC. Furthermore, individuals who are double heterozygotes for any combination of the two loci are also at high risk.

Data revealed that all the three SNPs contribute to CRC high-risk prediction through complex three-way interaction and rs9906859, particularly its heterozygous genotype, appears to be the most broadly and consistently associated with CRC high-risk. In addition, the predictive power of rs1558535 and rs17780195 is evident when they are in specific combinations, indicating that these three SNPs are crucial for precise risk stratification. Therefore, W-test and post-hoc epistasis analysis using 2-way interactions were performed to provides a more detailed breakdown of pairwise SNP associations with CRC.

MDR analysis identified the three-way model (Table 9) of LINC00511 rs1558535, rs17780195, and rs9906859 as the best predictive model of disease status, which minimized balanced accuracy in 5 out of 5 cross-validation intervals and estimates a prediction accuracy of 64.9% and classification accuracy of 66.1%. Data shown in Table 8 revealed that rs1558535 and rs9906859 are independently contribute to CRC risk, confirming data illustrated in Table 4. In addition, there are highly significant two-way interactions between all pairs of SNPs, and crucially, a highly significant three-way interaction among all three SNPs. This indicates that the risk of CRC is influenced by complex epistatic relationships between rs1558535, rs17780195, and rs9906859.

**Table 9:** W-Test of LINC00511 SNPs rs1558535, rs17780195, and rs9906859 Gene Interaction Analysis in colorectal cancer (n = 200) group and control (n = 200) group.

LINC00511 SNPs set	W	K	*P*
**Independent effects**			
rs1558535	14.4	3	< 0.01*
rs17780195	2.51	3	NS
rs9906859	18.1	3	< 0.001*
**Two-way interactions**			
rs1558535, rs17780195	79.7	9	< 0.001*
rs1558535, rs9906859	52.4	9	< 0.001*
rs17780195, rs9906859	23.5	8	0.001*
**Three-way interaction**			
rs1558535, rs17780195, rs9906859	50.2	19	< 0.001*

**Fig. 3:**
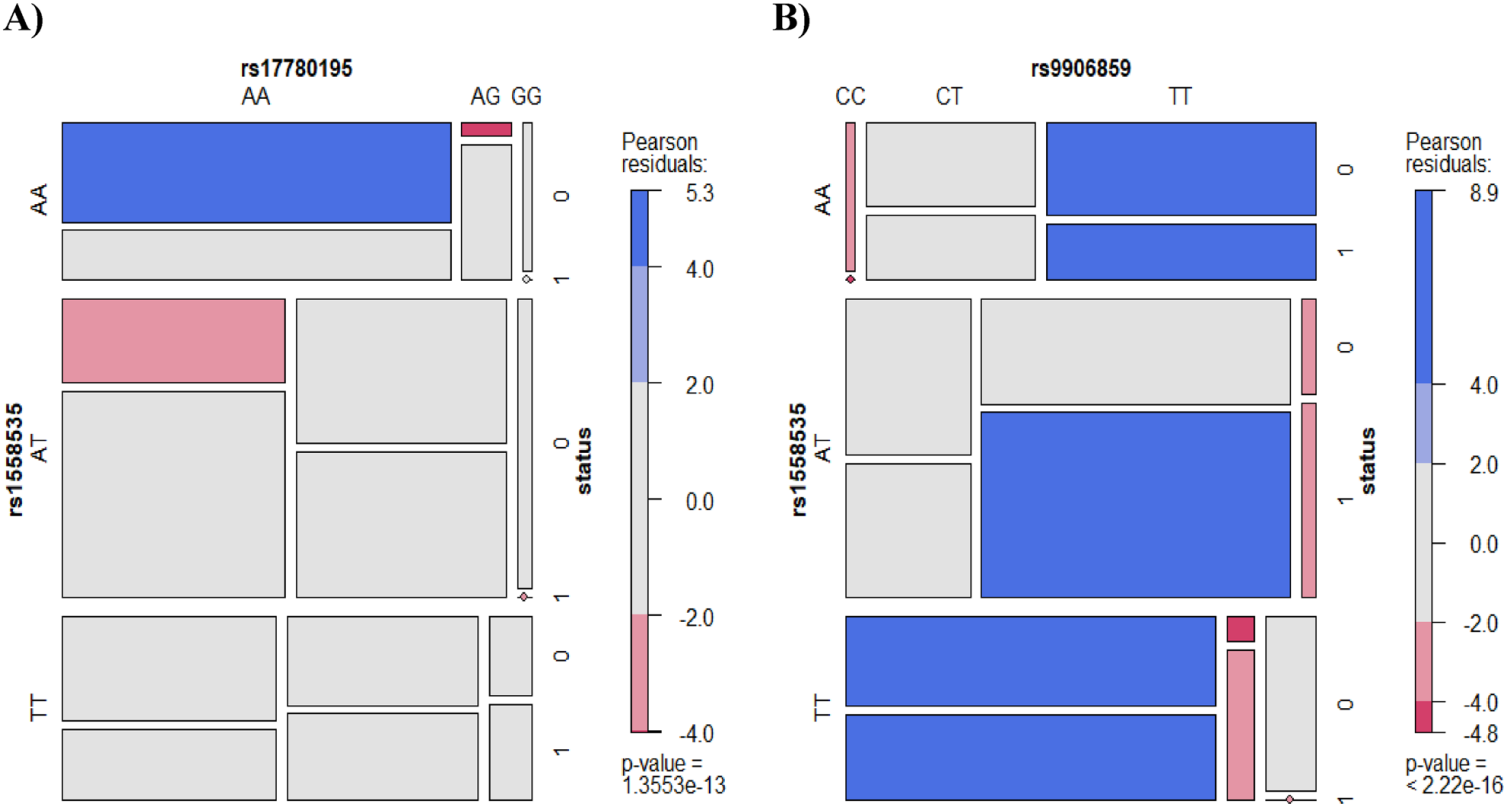
**Mosaic plot showing post-hoc epistasis analysis after multifactor dimensionality reduction (MDR) model fit with a two-way split for LINC00511 SNPs for CRC cases (n = 200) and controls (n = 200) (A)** *between* rs1558535 and rs17780195 **(B)** between rs1558535 and rs9906859. [Pearson Residuals; blue shading (positive residuals) indicates a positive association or enrichment, red shading (negative residuals) indicates a negative association, gray/white shading (near zero residuals) indicates that the observed counts are close to what would be expected under independence].

The mosaic plots (Fig. 3) that represent the post-hoc epistasis analysis display the relationship between (rs1558535 and rs17780195) (Fig. 3A) and (rs1558535 and rs9906859) (Fig. 3B). It is clear that there is a stronger statistically significant association between the later pair of SNPs and CRC, manifested by extremely low *P*-value. Certain genotype combinations such as (rs1558535 AT, rs9906859 TT) is significantly enriched in CRC patients, indicating their link to increased risk of CRC.

### Testing for linkage disequilibrium (LD) and pairwise correlation coefficient with haplotypes

CRC susceptibility would be influenced by the LD of neighbor genetic variations or linkage among other indirect SNPs, which may provide further insight into the connection of several non-random associated SNPs (haplotypes). A pair of alleles is said to be in linkage disequilibrium when they co-occur more frequently than would be expected based on their individual allelic frequencies. Linkage disequilibrium was assessed using the genotype data that had been gathered. Pairwise standardized linkage disequilibrium (D’) and haplotype analysis were calculated between each pair of LINC00511 polymorphism loci examined to evaluate the association between CRC susceptibility and LINC00511 SNPs rs17780195, rs9906859, and rs1558535.

**Fig. 4:**
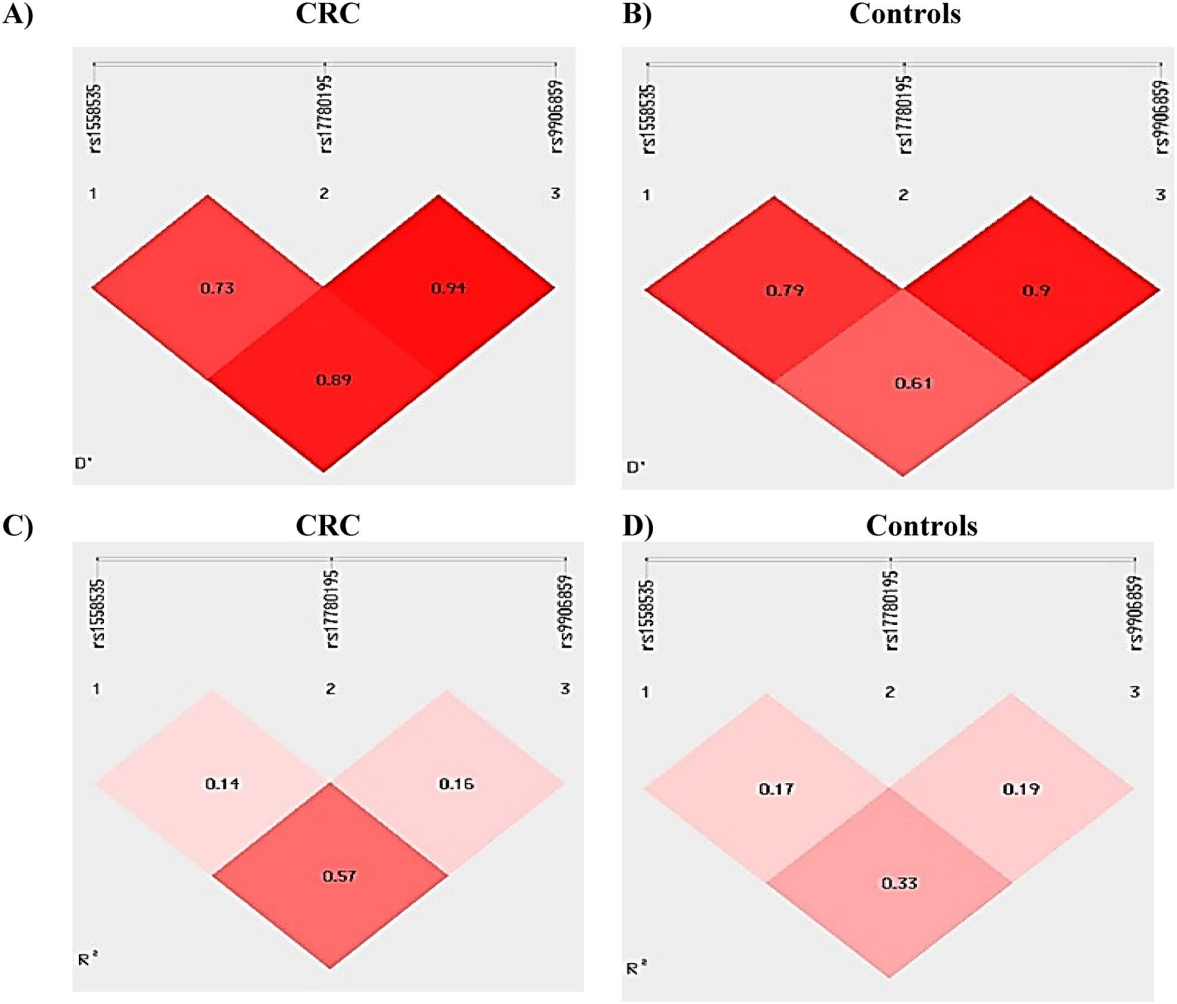
Haplotype block analysis between LINC00511 SNPs rs17780195, rs9906859, and rs1558535 A) calculated Pairwise standardized linkage disequilibrium D’ in pairs in CRC patients (n = 200) (left panel) and B) controls (n = 200) (right panel), C) calculated pairwise correlation coefficient R^2^ in pairs in CRC patients (n = 200) (left panel) and D) controls (n = 200) (right panel), [D’: Pairwise standardized linkage disequilibrium, R^2^: Correlation coefficient.].

Figure 4A shows a strong LD observed in the CRC group LINC00511 SNPs rs17780195 vs rs9906859 were highly linked according to the D’ (0.94), as well as the other two sets of pairwise loci LINC00511 SNPs rs9906859 vs rs1558535 D’ = 0.89, and finally, rs17780195 vs rs1558535 D’ = 0.73, exhibiting strong linkage as well. The most striking finding from comparing these two LD plots is LD between rs1558535 and rs9906859 in control group (Fig. 4B) that showed only moderate LD (D’ = 0.61) compared to their very strong LD (D’ = 0.89) in the CRC group. This suggests that a particular risk haplotype involving rs1558535 and rs9906859 might be more tightly linked and prevalent in individuals with CRC.

Pairwise correlation coefficient (*R*^*2*^) analysis (Figs. 4C and 4D) gave the same results as LD but with lesser extent. Greater correlation was observed between LINC00511 SNPs rs9906859 vs rs1558535 in CRC patients (R^2^ = 0.57) versus 0.33 in control group. Again, this difference indicates possible formation of a"risk-associated haplotype” involving rs1558535 and rs9906859 in CRC patients than in healthy controls. This comes in according with data shown in Table 7 considering ‘TAC’ mutant-wild-mutant haplotype for LINC00511 SNPs rs1558535, rs17780195, and rs9906859 as the haplotype with highest risk for CRC.

### Kaplan-meier survival analysis

Univariate analysis of the OS among the CRC patients (n = 200) without distant metastasis according to gender, tumor site, and late CRC stages are prognostic factors (Table 10).

**Table 10:** Univariate analysis of CRC patients’ (n = 200) survival and the known CRC prognostic clinicopathological factors.

Variable (Unit)	N = 200	OS HR (95% CI)	*P* value
**Gender**			
Male	61	1	
Female	139	5.1 (2–12.9)	0.001*
**BMI (kg/m**^**2**^**)**			
> 25	42	1	
≥ 25	158	0.72 (0.36–1.44)	NS
**Tumor site**			
Ascending	96	1	
Transverse	24	0.28 (0.11–0.75)	0.011*
Sigmoidal/Descending	80	2.34 (1.18–4.68)	0.015*
**Family history**			
No	150	1	
Yes	50	1.1 (0.512–2.395)	NS
**Tumor grade**			
T0	8	1	
T1	7	1979 (0.00–1.651E + 61)	NS
T2	17	7301.6 (0.00–5.909E + 61)	NS
T3	109	1035 (0.00–8.478E + 60)	NS
T4	59	1200 (0.00–9.826E + 60)	NS
**Lymph Nodes**			
N0	92	1	
N1	108	1.07 (0.39–2.95)	NS
**TNM stage**			
I	18	1	
II	71	0.795 (0.065–9.8)	NS
III	93	7.57 (0.524–109.35)	NS
IV	18	15.66 (1.147–213.71)	0.039*

* Statistical significance at 5% level. [BMI: Body mass index, CI: confidence interval, HR: hazard ratio, LINC00511: Long intergenic non-coding RNA 00,511, OS: overall survival, SNP: Single nucleotide polymorphism, NS: Non-significant].

**Fig. 5:**
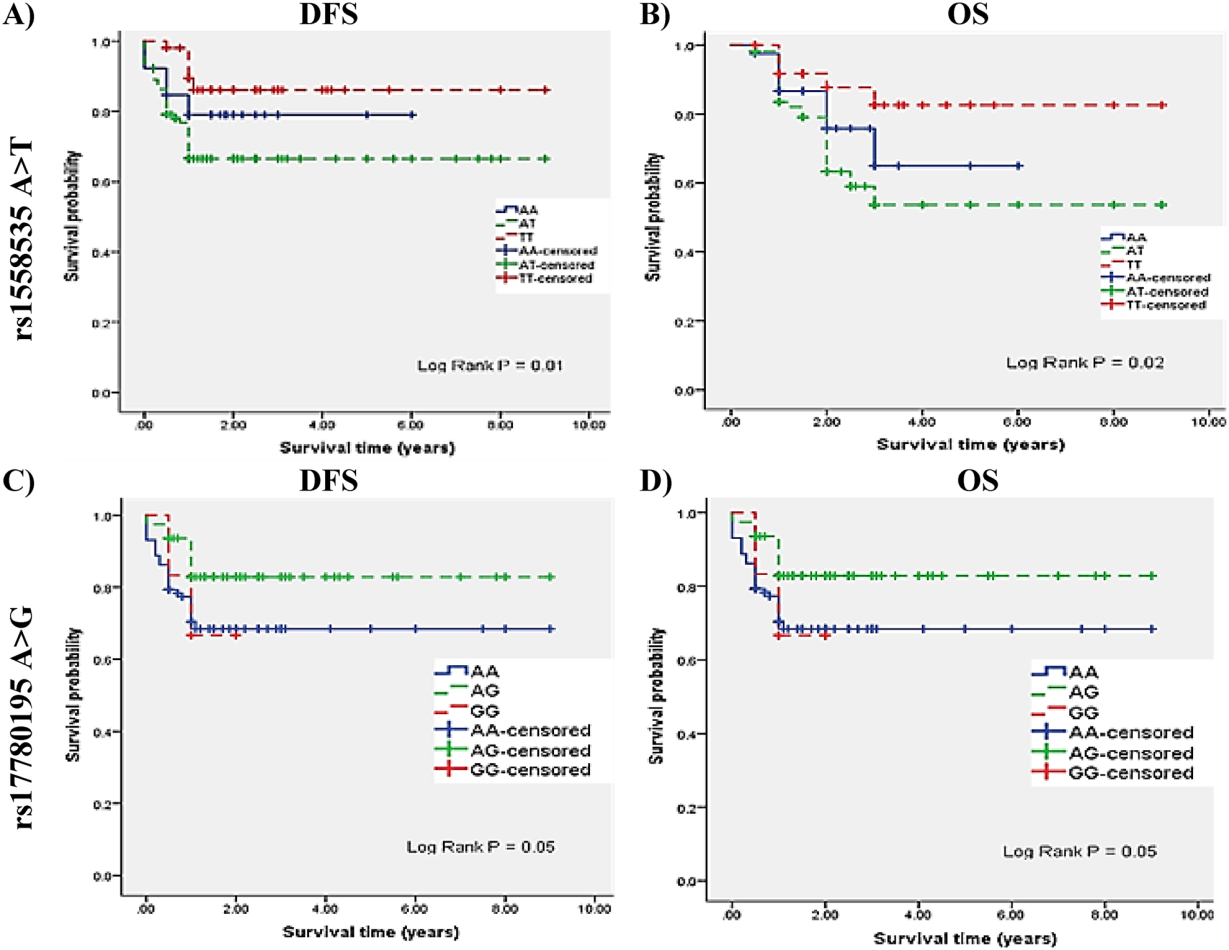
Kaplan–Meier survival analysis among CRC patients (n = 200) without distant metastasis according to LINC00511 SNPs genotypes A) DFS for rs1558535 A > T, B) OS for rs1558535 A > T, C) DFS for rs17780195 A > G and D) OS for rs17780195 A > G. [DFS, disease-free survival; OS, overall survival].

Kaplan–Meier survival analysis among the CRC patients (n = 200) without distant metastasis according to LINC00511 SNPs genotypes rs1558535 A > T DFS (Fig. 5A) and OS (Fig. 5B) and rs17780195 A > G DFS (Fig. 5C) and OS (Fig. 5D). However, for rs9906859 T > C DFS and OS were non-significant with P = 0.17 and P = 0.38, respectively.

## Discussion

CRC is one of the leading causes of cancer-related deaths worldwide^1,2^. There are more than approximately 1 million new cases of CRC globally^1,5^ with high mortality and poor prognosis. More research is required to identify potential cancer prognostic and predictive markers to mitigate mortality and improve prognosis^47^. Moreover, CRC is a multifactorial disease in which both genetic and epigenetic modification are highly implicated^8,14^.Genome association studies have highlighted the significant association between SNPs and different diseases^48,49^ including various cancer types^30,50,51^.

LncRNAs represent a new frontier in molecular biology, participating in the regulation of nearly every stage of gene expression^16,52^ and were found to be implicated in variety of cancers^18,53^. It is evident that only 7% of disease-associated SNPs are found within protein-coding regions while the rest 93% of SNPs are located within the non-coding regions^54^. In addition, molecular research has revealed significant links between CRC susceptibility and lncRNA SNPs^55–57^.

LINC00511 has been shown as oncogene linked to various cancers as breast and CRC^20,25,26^ and its genetic variants have been found previously to be associated with breast cancer in the Chinese population^28^. To the best of our knowledge, this is the first study to explore the association between LINC00511 SNPs and CRC risk, via elucidating LINC00511 SNPs (rs1558535, rs17780195 and rs9906859) *variants in* CRC patients’ liquid biopsy as well as to determine the implication of these SNPs prognostic performance in CRC Egyptian patients’ cohort.

This study revealed a prospective role of the 3 studied SNPs (rs1558535, rs17780195 and rs9906859) in mounting CRC risk. The results obtained revealed significant increased frequencies of heterozygous genotype of both rs1558535 A > T and rs9906859 T > C in CRC patients in comparison to controls. Also, carriers of the wild genotype of both rs1558535 and rs9906859 are much less in the patients’ group than the control subjects carrying such genotype. Unconditional logistic regression showed an independent correlation between rs1558535 and rs9906859 and CRC risk. The codominant regression model revealed that TC genotype of rs9906859 was associated with 3.54-fold increased risk in CRC, after adjustment for age and BMI. Dominant regression results depicted that rs9906859 TC + CC increased the risk of CRC by 3.04-fold. **This suggests “the strong predictive role of these genetic variants and CRC”.** The association between LINC00511 SNPs and the increased risk of CRC could be attributed to changes in the expression of the secondary structure of LINC00511.

Several studies have demonstrated that mutant variants can alter the secondary structure of lncRNAs, thereby affecting their interaction with microRNA (miRNA; miR) binding sites. LINC00511 could mediate tumorigenesis of CRC through sponging miRs such as miR-625-5p^25^ and miR-29c-3p^58^. Therefore, LINC00511 genetic variants could be implicated in CRC increased risk via interrupting the binding of LINC00511 with its target miRNAs and subsequently affecting the expression of downstream genes^1^.

In examining the association between tumor stage and grade with the three studied LINC00511 SNPs in the CRC patient cohort, none of the variants showed a significant correlation with tumor grade. However, a notable association was observed between certain SNP genotypes and advanced disease stages. Specifically, patients heterozygous for the mutant allele of rs1558535 were more frequently found in advanced CRC stages (III or IV) compared to early stages (I or II), with an odds ratio (OR) of 3.99, indicating a nearly fourfold increased likelihood of presenting with late-stage disease. Similarly, carriers of the rs17780195 AG genotype were significantly more likely to be diagnosed at stages III or IV, showing a 2.72-fold increase in the odds of advanced-stage CRC presentation compared to those with earlier-stage disease. These findings are clinically relevant, given that tumor stage is a well-established prognostic factor in CRC, with the 5-year survival rate dropping from approximately 64% in early-stage disease to just 12% in metastatic CRC^1,2,5^.. **Therefore, the strong association observed between tumor stage and both rs1558535 and rs17780195 variants in our CRC patient cohort suggests that these genetic variants may serve as potential prognostic biomarkers, given their link to CRC progression**. Furthermore, regression analysis identified several prognostic risk factors for CRC, including male gender, positive family history, and history of inflammatory bowel disease (IBD), all of which were significantly associated with higher tumor grade. Additionally, both tumor site and the presence of vascular infiltration were found to be significantly correlated with advanced CRC stages, further supporting their role in disease progression**.**

Haplotype analysis offers significant potential for disease gene mapping by leveraging the relationship between causative mutations and their ancestral haplotypes of origin. One of the most important findings in the current study is the detection of a significant correlation between LINC00511 SNPs haplotype and CRC. It was found that ‘T _rs1558535_ A _rs17780195_ C_rs9906859_’ ‘TAC’ as mutant-wild-mutant haplotype is associated with 1.5-fold CRC increased risk (OR: 1.46, 95% CI: 1.07–1.99). While ‘TAT’ haplotype conferred a fivefold lower CRC risk (OR: 0.20, 95% CI: 0.09–0.47). Gene interaction analysis and epistasis analysis showed that CRC patients who are all-heterozygous or are all-homozygous for the alternate allele in all studied loci are at CRC high risk. Furthermore, individuals who are double heterozygotes for any combination of the two loci are also at high risk. Additionally, individuals who carry at least one mutant allele (i.e. are heterozygote and homozygote or homozygote and heterozygote) at rs1558535 and rs9906859*,* respectively*,* are at high risk of CRC as well. *Even* individuals who are heterozygous at rs1558535 are also at high risk. This highlights **“the role of both rs1558535 and rs9906859 in predicting CRC risk”.**

Results of this study provided another level of evidence on the role of rs1558535 and rs9906859 in CRC risk. Result of one-way W-Test for gene interaction analysis revealed that either rs1558535 or rs9906859 is highly statistically significant independent predictors for CRC risk. In addition, two-way interaction W-test using different pairs of the studied SNPs showed high W-value of 52.4 for rs1558535 and rs9906859 pair, indicating a highly significant epistatic interaction between them. Moreover, the mosaic plot of post-hoc epistasis analysis visualized the strong and complex interplay between these genotypes in determining CRC risk. Some genotype combinations between this pair of SNPs especially (rs1558535 AT, rs9906859 TT) genotype found to significantly enriched in CRC patients than control, underscoring how these two SNPs might influence an individual’s susceptibility to CRC.

This would be further confirmed or rolled out by LD analysis of neighbor genetic variants to assess the connection of several haplotypes. In this study, pairwise LD analysis showed a strong association in CRC group between different pairs (rs1558535 and rs9906859) as well as (rs17780195 and rs9906859). The most notable difference is the substantially stronger LD between rs1558535 and rs9906859 in the CRC group compared to the control group (D’ = 0.89 vs 0.61, respectively). This suggests that a specific haplotype combination involving these two SNPs is preferentially preserved and more prevalent in CRC patients. In the same line, correlation analysis revealed the same results. Greater correlation was observed between LINC00511 SNPs rs1558535 and rs9906859 in CRC patients than in the control group (R^2^ = 0.57 in CRC vs 0.33 in controls). This implies that certain alleles at these two loci might be preferentially combined together in CRC patients forming a"risk-associated haplotype". These finding provide further insights into **“the connections of the studied SNPs, particularly rs1558535 and rs9906859 and their significant association with CRC susceptibility”.**

Finally, we analyzed the OS and DFS in CRC group. The univariate analysis revealed that the late CRC stage IV showed 15.66-fold increase in the OS hazard ratio (OR: 15.66, 95%CI: 1.147–213.71). This is consistent with previous research considering advanced CRC stage, and its related factors, as strong predictor(s) of poor CRC prognosis/outcome^1,5,7^.

**Shortcomings to this study** were missing data for the genotypes for some patients, decreasing the statistical efficiency.

### Strength(s) within the present study

First, the study included a relatively adequate sample size and was conducted on a population that represents a common and relevant demographic, enhancing its generalizability. Second, confounding bias was minimized through frequency matching of cases and controls. Third, the study is a step-toward personalized and precision medicine application, determine the genetic variants in lncRNA SNPs “implicating-to-disease”, aligning with the objectives of broader epigenomic and genomic research initiatives. Fourth, as part of the study’s **future perspective**, 10% of the samples will be selected for validation of genotyping results using lncRNA sequencing (lncRNA-seq) to confirm and expand upon current findings**.** Moreover, two ongoing researches, by our research group, at the Advanced Biochemistry Research Lab, Faculty of Pharmacy, Ain Shams University, are addressing the same LINC00511 SNPs variants haplotype role in breast cancer Egyptian female patients as well as hepatocellular carcinoma Egyptian patients’ prognosis and pathogenesis.

### Sustainability plan

First, lncRNA-seq. via next generation sequencing (NGS) to study functional roles in diverse biological processes and human diseases, such as cancer, using the mutant samples. Second, LINC00511 neighbor SNPs prediction (rs4432291) using genotype imputation technique by the relevant possible software as MACH, IMPUTE, BEAGLE, SNPTEST (used for genotype imputation uncertainty when performing a test for association between genotypes and phenotypes) or BIMBAM and PLINK (the free open-source program) used as GWA analysis toolset^59^. Third, the OS and DFS are to be correlated with LINC00511 SNPs variants and lncRNA-seq. results.

## Conclusion(s)

In brief, LINC00511 SNPs (rs1558535, rs17780195 and rs9906859) are associated with CRC increased risk. LINC00511 SNPs rs1558535 and rs17780195 are highly linked to CRC late stages. Individuals who are TT/AG/TC or AT/AG/CC for the alternate allele in all studied loci of SNPs rs1558535 and rs9906859 are at high risk of CRC.

## Supplementary Information

Supplementary Information.

## Data Availability

Data is provided within the manuscript or supplementary information files.

## Abbreviations

AUC: Area-under-receiver
BMI: Body mass index
ceRNA: Competitive endogenous RNA
CI: Confidence interval
CRC: Colorectal cancer
DFS: Disease-free survival
EFS: Event free survival
HR: Hazard ratio
HW: Hardy–weinberg equilibrium
IBD: Inflammatory bowel disease
IQR: Interquartile range
LD: Linkage disequilibrium
LINC00511: Long intergenic non-coding RNA 00511
LncRNAs: Long non-coding RNAs
MAF: Minor allele frequency
MDR: Multifactor dimensionality reduction
miRNA/miR: MicroRNA
ncRNAs: Non-coding RNAs
NPP: Negative predicted value
OR: Odds ratio
OS: Overall survival
PPV: Positive predictive value
Rs: Reference SNP
RT-PCR: Real-time polymerase chain reaction
SN: Sensitivity
SNPs: Single nucleotide polymorphisms
SP: Specificity
TMs: Tumor markers
TNM: Tumor-node-metastasis
