# Supplementary material for: Long intergenic non-coding RNA 00511 (LINC00511) genetic variations and haplotype implication for colorectal cancer susceptibility and prognosis
Source: Sci Rep. 2025 Aug 11;15:29388. doi: 10.1038/s41598-025-10938-7 (PMC12339720; doi:10.1038/s41598-025-10938-7)
Supplement: Supplementary file 1 — Supplementary Information. [file 41598_2025_10938_MOESM1_ESM.docx]

**Title Page:**

**Article Type:** Research Article

**Oncogenic LINC00511 Genetic Variations and Haplotype Implication for CRC Susceptibility and Survival Case-Controlled Study; a Step toward ncRNA Precision**

**Eman F. Sanad^1,*^,** Ahmed Al-Hady^2^, Mohamed Abo-Auf Ali^3^, Shorouk Eldash^4^, Nermeen H. Elmorshedy^5^, Farah Ayman^5^, Hams M. Khattab^5^, Amr Maher^5^, Hadeel Ashree^5^, Mahitab Abdelhady^5^, Mazen Mohamed^5^, Ahmed Adel^5^, Sajed Khalil^5^, Omar Alyan^5^, Ahmed Samir^5^, Alhassan A. Bakr^5^, **Nadia M. Hamdy^1,*^**

***Corresponding Authors:**

[dr.emansanad@pharma.asu.edu.eg](mailto:dr.emansanad@pharma.asu.edu.eg) orcid.org/0000-0001-5406-2029

[nadia_hamdy@pharma.asu.edu.eg](mailto:nadia_hamdy@pharma.asu.edu.eg) orcid.org/0000-0003-2105-107X

**Table 1S:** **Model Fit and predictive performance Measures for the codominant, dominant, over-dominant, and recessive models**

| **Model** | **AUC** | **DIC** | **AIC** | **BIC** | **R²_McF_** | **R²_CS_** | **R²_N_** | **χ²** | **df** | ***P*^*^** |
| --- | --- | --- | --- | --- | --- | --- | --- | --- | --- | --- |
| **Codominant** | 0.786 | 446 | 464 | 500 | 0.196 | 0.238 | 0.317 | 108 | 8 | < .001 |
| **Dominant** | 0.761 | 457 | 469 | 493 | 0.175 | 0.216 | 0.287 | 97.1 | 5 | < .001 |
| **Over-dominant** | 0.784 | 449 | 461 | 485 | 0.190 | 0.232 | 0.309 | 105 | 5 | < .001 |
| **Recessive** | 0.750 | 465 | 477 | 501 | 0.161 | 0.200 | 0.267 | 89.2 | 5 | < .001 |

*Omnibus Likelihood Ratio Test. [AIC: Akaike Information Criterion, AUC: Area-Under-receiver-operating characteristic Curve, BIC: Bayesian Information Criterion, df: Degrees of freedom, DIC: Deviance Information Criterion, R²_CS:_ Cox and Snell’s Pseudo R^2^, R²_McF_: McFadden’s R^2^, R²_N_: Nagelkerke’s R^2^.]

**Table 2S: Associations between LINC00511 SNPs rs1558535, rs17780195, rs9906859 genotypes, and colorectal cancer in the co-dominant, dominant, over-dominant, and recessive genetic models**

| **Characteristic** | **Model** | **Genotype** | **Case**, N (%) | **Control**, N (%) | **P^*^** | **OR^**^** | **95% CI** | **P** |
| --- | --- | --- | --- | --- | --- | --- | --- | --- |
| rs1558535 A>T | Codominant |  |  |  | **0.002** |  |  |  |
|  |  | AA | 39 / 200 (20) | 60 / 200 (30) | **0.020** | 1.00 | — |  |
|  |  | AT | 111 / 200 (56) | 78 / 200 (39) | **< 0.001** | 1.52 | 0.72—3.21 | NS |
|  |  | TT | 50 / 200 (25) | 62 / 200 (31) |  | 1.32 | 0.53—3.28 | NS |
|  | Dominant |  |  |  | **0.015** |  |  |  |
|  |  | AA | 38 / 200 (19) | 60 / 200 (30) |  | 1.00 | — |  |
|  |  | AT + TT | 162 / 200 (81) | 140 / 200 (70) |  | 1.13 | 0.56—2.25 | NS |
|  | Over-dominant |  |  |  | **< 0.001** |  |  |  |
|  |  | AA + TT | 90 / 200 (45) | 123 / 200 (62) |  | 1.00 | — |  |
|  |  | AT | 110 / 200 (55) | 77 / 200 (38) |  | 1.33 | 0.78—2.27 | NS |
|  | Recessive |  |  |  | NS |  |  |  |
|  |  | AA + AT | 148 / 200 (74) | 137 / 200 (68) |  | 1.00 | — |  |
|  |  | TT | 52 / 200 (26) | 63 / 200 (32) |  | 0.75 | 0.40—1.39 | NS |
| rs17780195 A>G | Codominant |  |  |  | NS |  |  |  |
|  |  | AA | 116 / 200 (58) | 117 / 200 (58) | NS | 1.00 | — |  |
|  |  | AG | 78 / 200 (39) | 70 / 200 (35) | NS | 0.78 | 0.46—1.32 | NS |
|  |  | GG | 6 / 200 (3.0) | 13 / 200 (6.5) | NS | 0.56 | 0.16—1.82 | NS |
|  | Dominant |  |  |  | NS |  |  |  |
|  |  | AA | 116 / 200 (58) | 117 / 200 (58) |  | 1.00 | — |  |
|  |  | AG + GG | 84 / 200 (42) | 83 / 200 (42) |  | 0.67 | 0.41—1.11 | NS |
|  | Over-dominant |  |  |  | NS |  |  |  |
|  |  | AA + GG | 122 / 200 (61) | 130 / 200 (65) |  | 1.00 | — |  |
|  |  | AG | 78 / 200 (39) | 70 / 200 (35) |  | 0.96 | 0.59—1.55 | NS |
|  | Recessive |  |  |  | NS |  |  |  |
|  |  | AA + AG | 194 / 200 (97) | 187 / 200 (94) |  | 1.00 | — |  |
|  |  | GG | 6 / 200 (3.0) | 13 / 200 (6.5) |  | 0.56 | 0.17—1.71 | NS |
| rs9906859 T>C | Codominant |  |  |  | < 0.001 |  |  |  |
|  |  | TT | 26 / 200 (13) | 52 / 200 (26) | 0.002 | 1.00 | — |  |
|  |  | TC | 104 / 200 (52) | 69 / 200 (34) | 0.001 | 3.54 | 1.58—8.13 | .002 |
|  |  | CC | 70 / 200 (35) | 79 / 200 (40) | NS | 1.55 | 0.61—4.02 | NS |
|  | Dominant |  |  |  | 0.002 |  |  |  |
|  |  | TT | 26 / 200 (13) | 52 / 200 (26) |  | 1.00 | — |  |
|  |  | TC + CC | 174 / 200 (87) | 148 / 200 (74) |  | 3.04 | 1.43—6.64 | .004 |
|  | Over-dominant |  |  |  | < 0.001 |  |  |  |
|  |  | TT + CC | 96 / 200 (48) | 131 / 200 (66) |  | 1.00 | — |  |
|  |  | TC | 104 / 200 (52) | 69 / 200 (34) |  | 2.57 | 1.49—4.52 | 0.001 |
|  | Recessive |  |  |  | NS |  |  |  |
|  |  | TT + TC | 130 / 200 (65) | 121 / 200 (60) |  | 1.00 | — |  |
|  |  | CC | 70 / 200 (35) | 79 / 200 (40) |  | 1.36 | 0.74—2.48 | NS |

## **P*-values for post-hoc comparisons in the co-dominant model are adjusted for multiple testing per the Benjamini and Hochberg procedure.

## **Odds ratios are obtained by logistic regression analysis adjusted for age and BMI. Age, CEA, and CA19-9 optimal cut-offs for disease prediction were determined using receiver operating characteristics analysis. [BMI: Body-Mass Index, LINC00511: Long Intergenic Non-Protein Coding RNA 51
